# Prediction of pharmacological activities from chemical structures with graph convolutional neural networks

**DOI:** 10.1038/s41598-020-80113-7

**Published:** 2021-01-12

**Authors:** Miyuki Sakai, Kazuki Nagayasu, Norihiro Shibui, Chihiro Andoh, Kaito Takayama, Hisashi Shirakawa, Shuji Kaneko

**Affiliations:** 1 Department of Molecular Pharmacology, Graduate School of Pharmaceutical Sciences, Kyoto University, 46-29 Yoshida-Shimoadachi-cho, Sakyo-ku, Kyoto, 606-8501 Japan; 2 Medical Database Ltd., 2-5-5 Sumitomoshibadaimon building, Shibadaimon, Minato-ku, Tokyo, 105-0012 Japan

**Keywords:** Drug safety, Drug screening, Pharmaceutics, Pharmacology, Toxicology, Data mining, High-throughput screening, Machine learning, Virtual drug screening, Transporters in the nervous system, Neuroscience

## Abstract

Many therapeutic drugs are compounds that can be represented by simple chemical structures, which contain important determinants of affinity at the site of action. Recently, graph convolutional neural network (GCN) models have exhibited excellent results in classifying the activity of such compounds. For models that make quantitative predictions of activity, more complex information has been utilized, such as the three-dimensional structures of compounds and the amino acid sequences of their respective target proteins. As another approach, we hypothesized that if sufficient experimental data were available and there were enough nodes in hidden layers, a simple compound representation would quantitatively predict activity with satisfactory accuracy. In this study, we report that GCN models constructed solely from the two-dimensional structural information of compounds demonstrated a high degree of activity predictability against 127 diverse targets from the ChEMBL database. Using the information entropy as a metric, we also show that the structural diversity had less effect on the prediction performance. Finally, we report that virtual screening using the constructed model identified a new serotonin transporter inhibitor with activity comparable to that of a marketed drug in vitro and exhibited antidepressant effects in behavioural studies.

## Introduction

The pharmacological actions of drugs are dependent on their binding affinity to specific target proteins. It is normally impossible to predict in advance how strongly an individual compound will act on a target protein by looking only at their structures, even for the most experienced researchers. This activity prediction problem has been studied in the field of cheminformatics for many years, and is now a central component of drug discovery due to rapid progress in, first, in silico screening and, more recently, machine learning. One relevant machine learning technology is a deep neural network (DNN). The convolution technique in a DNN is a core element of the revolutionary capabilities of computer vision, which has attracted ever-increasing attention to DNNs^1^. When this technique is applied to a chemical compound, structural information is converted into a numerical form, a feature vector, which can be machine processed to explain the relationship between that compound and its pharmacological activity.

Graph convolutional neural network (GCN) models that combine neural fingerprints with fully connected layers show improved performance in tasks such as solubility prediction and activity prediction compared with extended-connectivity circular fingerprint (ECFP)-based models, which are one of the standard methods of compound representation^2,3^.

Altae et al. reported a GCN model that defines a new layer, similar to the pooling layer used in image recognition tasks, and a graph gathering layer; these layers are available for research under an open-source license as a central part of the DeepChem application^4,5^. They compared the classification performance of their GCN model with the support vector machine (SVM) model, which is a method commonly used in machine learning, for the Tox 21 (toxicity), SIDER (adverse event), and MUV (pharmacological activity) datasets. That study demonstrated that GCN models can exhibit performance comparable to or better than that of SVMs even without “thorough hyperparameter optimization” of the GCN models.

With the help of this easy-to-use open-source algorithm, much successful classification performance has been reported. A GCN architecture with one fewer convolutional layer than Altae’s classified the inhibitory activity of compounds against the human ether-a-go-go-related gene (hERG; a risk factor for severe cardiac arrhythmia)^6^ and the bioactivity of per- and polyfluorinated alkyl substances^7^, and showed that the GCN models outperformed nine other machine learning techniques for the datasets in MoleculeNet^3^. Another GCN architecture with the same three convolutional layers as Altae’s successfully classified compounds for 10 targets extracted from the PubChemBioAssay collection^8^ and compounds that act on β-site amyloid precursor protein cleavage enzyme 1 (BACE1; a major drug target in Alzheimer’s disease)^9^. Mayr et al. extensively validated the performance of nine types of classification models, including GCNs, for 1310 assays collected from ChEMBL (release 20), a database of bioactive molecules with drug-like properties^10^.

The objective variable in classification is one or multiple binary values. The thresholds required for defining an active (or inactive) compound should vary depending on the target being addressed, however, no fixed rules have been observed^10–12^. In addition to the threshold setting, there is another problem of losing crucial information about the “degree” of binding to the targets. For instance, a compound with an IC_50_ value of 1 μM and a compound with an IC_50_ value of 1 nM are equally treated as “active” in a classification task, although the latter compound is obviously far more potent than the former under the same experimental setting.

In early drug discovery research, high-throughput screening is an important source of information, and quantitative outcomes are more valuable than simple qualitative data for selecting the compounds to be optimized. Similarly, when purchasing a limited number of compounds from a large virtual compound library, for example, quantitative activity predictions will make the prioritizing process easier. Furthermore, to identify tool compounds to elucidate pharmacological actions, quantitative predictions will be more helpful than qualitative predictions. To this end, lines of reports have constructed various regression models using chemical representations in conjunction with information on their targets, such as three-dimensional compound-protein complex information^13^, amino acid sequence information^14–16^, assay information for target proteins^17,18^, and information on the atoms from the amino acid in the vicinity of the binding site of a compound^19,20^.

By contrast, regression models using only compound-derived data have also been reported. One used a feature vector transformed from very long ECFPs of up to 102,400 bits to predict the activity of G protein-coupled receptor (GPCR) ligands^21^. Another used a composite feature vector generated by concatenating two types of fingerprints (neural fingerprints and conventional fingerprints) to predict the activity for targets where the protein–ligand complex structure had been solved^22^. Quantitative activity prediction seeks to predict an infinite variety of objective variables. Since architectures with many nodes in the hidden layer perform better even for activity classification^12,23^, more nodes are required in quantitative prediction.

Many drugs are compounds that are easily described by simple chemical structures, which themselves contain the key determinants of their pharmacological actions. A compound of this kind is capable of taking various conformations depending on the number of its degrees of freedom, but in many cases, its preferred conformation is inherent to the chemical structure itself, although only specific conformations are normally involved in its pharmacological mode of action. Moreover, a drug must also be absorbed and reach the site of action. The physicochemical properties behind drug absorption and distribution are also essential features of its chemical structure.

In this paper, we report the performance of regression models built only on features that are automatically extracted from compound structures. Specifically, taking a chemical structure as a graph, we construct GCN models and show that the models with larger hidden layers satisfactorily and quantitatively predict the half-maximum responses of publicly available measures, IC_50_, EC_50_, K_i_, K_d_, and K_m_. By building models for a benchmark dataset of 127 target proteins extracted from the ChEMBL release 25 (referred to as ChEMBL in this report) and by using an information theory metric introduced in this study, we demonstrate that the diversity of compound structures in the dataset had less impact on the predictive performance than expected. We also report that our model identified a new compound via virtual screening of the serotonin transporter (SERT), whose binding capacity is comparable to that of a commercial drug in an in vitro assay and antidepressant effects in in vivo assays.

## Materials and methods

### Dataset

Data were extracted from ChEMBL by adjusting the protocol of Bosc et al.^11^. First, data with confidence scores of 6 or greater, assay type = B, and standard units = nM were selected. These confidence scores were provided by ChEMBL and indicate the level of confidence in the target protein assignment to the compound. B indicates a “binding” assay by an in vitro experiment. For each target, p-activity values were used throughout this study; these are defined by − log (*v*) and referred to as pIC_50_. In this context, *v* is one of IC_50_, EC_50_, K_i_, K_d_, and K_m_, where higher values indicate greater activity. The standard relation was chosen to be one of “>”, “≥”, “=”, “≤”, and “<”. As a further limitation, if the “activity_comment” was neither “Inconclusive” nor “Not determined” and if the “potential duplicates” = 0 and “data_validity_comment” was anything but “Potential author error”, the measurements were selected.

Compound structures were extracted from ChEMBL in SMILES format (simplified molecular input line entry system). They were neutralized with Instant J Chem 19.8.0 (IJC)^24^, solvents and salts were removed according to the built-in dictionary, descriptions of some functional groups were standardized, and finally, they were converted to canonical SMILES. Note that only SMILES with a length of 1000 or fewer were used in this study (default setting of IJC). For a compound with more than one tautomer, it was assumed that the most reasonable one was registered in ChEMBL, and it was used as provided. When a compound-target pair had multiple pIC_50_ values, the maximum (= most active) value was adopted.

### Data splitting

For each target, the dataset was randomly divided into two subsets, a training-validation set (90%) and a test set (10%). The training-validation set was further divided into a training set (88.8%) and a validation set (11.2%). The ratio of the sizes of these three subsets after the split was approximately 80:10:10.

### Graph convolutional neural network

First, each canonical SMILES was transformed into a binary vector of 75 dimensions per atom by RDKit^25^ implemented in DeepChem (default setting of DeepChem). These vectors consisted of physicochemical properties, such as the atomic type, number of valences, formal charges, and hybridization (Supplementary Table S1). Briefly, using the initial vector as input, the information of neighbouring atoms was added in the graph convolutional layer, and the information of the atoms was updated with the maximum value in the neighbouring atoms in the graph pooling layer. After this operation was repeated, the vector was converted into one dense layer. The numerical vectors represented by the dense layer were added together in the graph gathering layer to generate the “neural fingerprint” of the compound. The graph gathering layer was fully connected to an output layer of one neuron, and the entire network was trained to minimize the loss function so that each output layer reproduced its corresponding pIC_50_ (Supplementary Fig. S1). Adam was used as the optimization method, ReLU (convolutional layer) and tanh (graph gathering layer) were used as activation functions, and batch normalization was applied to prevent overfitting and improve the learning efficiency.

### Hyperparameter optimization and model training

To optimize the hyperparameters, Bayesian optimization with Gaussian processes was applied via the pyGPGO package^26^ and DeepChem 2.1.0 throughout this study. In the GCN architecture, two to four convolutional layers have been primarily used^5–10^. On the other hand, in our preliminary experiments, we found that a “shallow” network architecture with one convolutional layer performed better than a “deep” (two or more layers) architecture. Furthermore, the preliminary results indicated that an appropriate number of convolutional layers was four at the maximum, and having additional convolutional layers hindered the prediction ability. Based on these observations, the hyperparameters were explored independently for architectures with one, two, and three to four convolutional layers. A Bayesian optimization search was performed 100 times with the Matérn kernel as a covariance function and “expected improvement” as an acquisition function. This calculation was repeated four times with different weights initialized by a random seed value. In the case of small datasets used to examine the effect of the dataset size on model performance, a limited parameter range was applied.

In quantitative activity prediction, the mean absolute error (MAE), root-mean-square error (RMSE), and coefficient of determination (R^2^) are widely used as statistical metrics of model performance and are calculated by Eqs. (1)–(5).

1 $${\text{MAE }} = \frac{1}{n}\mathop \sum \limits_{i = 1}^{n} \left| {f_{i} - y_{i} } \right|$$

2 $${\text{RMSE }} = \sqrt {\frac{1}{n}\mathop \sum \limits_{i = 1}^{n} \left( {f_{i} - y_{i} } \right)^{2}}$$

3 $${\text{R}}^{{2}} = { 1 }{-} \, \left( {{\text{RSS}}/{\text{TSS}}} \right)$$

4 $${\text{TSS }}\left( {\text{total sum of squares}} \right){ } = \mathop \sum \limits_{i = 1}^{n} \left( {y_{i} - y} \right)^{2}$$

5 $${\text{RSS }}\left( {\text{residual sum of squares}} \right){ } = \mathop \sum \limits_{i = 1}^{n} \left( {y_{i} - f_{i} } \right)^{2}$$

where ${y}_{i}$ and ${f}_{i}$ represent the reported and predicted *i*th compound activity, $y$ is the average of ${y}_{i}$, and $n$ is the number of compounds. We evaluated the hyperparameter settings using the MAE and a new metric (2R2_MAE) defined in Eq. (6).

6 $${\text{2R2}}\_{\text{MAE }} = \, \left( {{\text{R}}^{{2}} - {\text{ MAE}}} \right)+{\text{R}}^{{2}}$$

2R2_MAE is based on the simple idea below; the higher its value is, the better.

1. For parameter settings that give the same MAE, a higher R^2^ value is better. (This is represented by R^2^ − MAE, the first term).
2. If the first term has the same value among parameter sets, a set with a higher R^2^ value is better (R^2^ is the second term).

From a set of 100 hyperparameters obtained after 100 iterations of Bayesian optimization search to minimize the MAE values, one hyperparameter set with a maximal 2R2_MAE value for the held-out validation set was selected, and finally, four hyperparameter sets were obtained for each network architecture. Since a “shallow” network architecture tended to give better R^2^ values than a “deep” network architecture, we re-ran 1000 hyperparameter search calculations if all R^2^ values for networks with one convolutional layer were lower than 0.45. For networks with two convolutional layers, the hyperparameter set with an R^2^ value of 0.40 or more was retained. For much deeper networks, the hyperparameter set was retained when its R^2^ value was higher than any R^2^ value of the shallower networks.

The final model training was performed on the best hyperparameter set (excluding epochs) with a fixed initial seed. For each model, 100 epochs were first calculated. If the minimum MAE on the held-out validation set did not decrease further in the next 100 epochs, the learning was terminated. When the MAE value decreased, another 100 epochs of learning were conducted, and the same procedure was repeated without setting an upper limit for the total number of epochs until the previous minimum MAE no longer changed during the additional 100 epochs. After the learning, a 2R2_MAE value was calculated for each epoch, and a model with the maximum 2R2_MAE value was selected as a final model. Final models were built using DeepChem 1.3.0. The graph convolution algorithms implemented in DeepChem 1.3.0 and 2.1.0 used for hyperparameter search are the same.

### Ensemble learning

Ensemble learning is a common technique in machine learning, where multiple models are constructed and combined. Many studies have shown that ensemble learning improves prediction accuracy compared to individual models^23,27–29^. We applied this technique by simply averaging the individual outputs without weighting. In this report, the predicted pIC_50_ values refer to the output of ensemble learning, unless otherwise noted.

### Scaffold diversity

Considering that the structural diversity of a dataset is one of the factors affecting the prediction performance and generalizability of models, we assessed the distribution of Murcko scaffolds^30^ in ChEMBL by removing all side chains of compounds and replacing all heavy atoms with carbons. By adapting Shannon's definition used in information theory, the quantitative scaffold diversity index (H) was introduced as Eq. (7).

7 $$\mathrm{H }=-\sum {p}_{i}{{log}_{2}p}_{i}$$

In this formulation, ${p}_{i}$ is the fraction of the number of compounds (${c}_{i}$) containing a certain scaffold relative to the total number ($c$) of compounds.

8 $${{p}_{i}=c}_{i}/c$$

A smaller H value means that the distribution is more biased towards particular scaffolds, while the maximum value is obtained for a uniform distribution. With IJC, 145,515 scaffolds were found in ChEMBL. For each dataset, the scaffolds were sorted in ascending order by scaffold size (the number of carbons that make up a scaffold), transformed to a histogram containing 10,000 scaffolds per bin, and converted to a probability distribution by dividing the number of compounds in each bin by the total number of compounds in the dataset (Note that the 15th bin has only 5515 scaffolds). Since there is no reasonable number of bins, we used 15 bins throughout this study, referring to previous reports^31,32^. For 15 bins, H has a maximum value of 3.91 (H_max_ = ${log}_{2}(15)$ = 3.91).

In addition to H, we employed the Kullback–Leibler divergence (KLD) as a metric to quantify the difference in the scaffold distributions between datasets before and after the random split.

9 $$\mathrm{KLD }= -\sum {p}_{i}{log}_{2}({p}_{i}/{q}_{i})$$

where ${q}_{i}$ is the probability distribution of the scaffolds in an unsplit dataset and ${p}_{i}$ is the probability distribution of the training set, validation set, or test set. KLD is always non-negative, and a minimum of zero is obtained when ${q}_{i}= {p}_{i}$. The same histograms used for the H calculations were also used to calculate the KLD.

## Materials

Citalopram and CHEMBL1377753 (5-chloro-2-(piperidin-4-yl)-1,3-benzothiazole hydrochloride, **1**) were purchased from Namiki Shoji (Tokyo, Japan). For the in vivo assay, **1** was dissolved in saline just before use. For the in vitro assay, citalopram and **1** were dissolved in Hank’s balanced salt solution (HBSS; Thermo Fisher Scientific, Waltham, MA, USA) and stored at − 20 °C until use.

### SERT substrate uptake assays in HEK cells

IC_50_ determinations were performed using the Neurotransmitter Transporter Uptake Assay Kit (R8173, Molecular Devices, San Jose, CA, USA) according to the manufacturer’s instructions and previous reports^33^. Briefly, HEK293 cells were seeded on 96-well black-wall clear-bottom plates (#655090, Greiner, Kremsmünster, Austria) at a density of 3.85 × 10^4^ cells/well. The cells were transfected with plasmid DNA (hSERT-pcDNA3 (Addgene #15483^34^) or pcDNA3; 200 ng/well) using Lipofectamine 2000 (Thermo Fisher Scientific). After 28–30 h of incubation, the cells were directly used for IC_50_ determination. For IC_50_ determination, the culture medium was changed to HBSS. Then, HBSS-containing drugs and HBSS-containing dye were sequentially added to the culture. After 60 min of incubation, the fluorescence was measured by a Wallac 1420 ARVOsx multilabel counter (Perkin Elmer, Waltham, MA, USA). The background was defined as the fluorescence of the pcDNA3-transfected well containing each concentration of drug to mitigate the effect of the possible fluorescence of the applied drugs. The specific uptake was defined as the fluorescence of each hSERT-transfected well subtracted by the corresponding background. The specific uptake was normalized to that in the absence of a drug. The IC_50_ values were calculated using Prism 8 (GraphPad Software, San Diego, CA, USA; https://www.graphpad.com/scientific-software/prism/).

### Animals

All animal care and experimental procedures were approved by the Kyoto University Animal Research Committee (Approval number 19-41) and performed following the ethical guidelines of the Committee. Adult male C57BL/6J mice (8–16 weeks old, 22–28 g body weight. Nihon SLC, Shizuoka, Japan) were housed in groups (no more than 6 mice in an individual cage) with free access to food and water and kept under constant ambient temperature (24 ± 1 °C) and humidity (55 ± 10%), with a 12-h light–dark cycle. Animals were randomly assigned to each experimental group. All behavioural tests were performed in the light cycle of the day.

### Behavioural tests

All behavioural tests were performed and analysed by experimenters who were blind to the injected drugs. The tail suspension test was performed as previously described^35^. Briefly, after acclimation, mice were hung on a hook (35 cm from the floor of the test box) with the tail taped to a force transducer (PowerLab 2/26, AD Instruments, Dunedin, New Zealand) fixed to the ceiling of the test box (40 × 40 × 40 cm). The immobility time was recorded for 6 min. Administration of each drug was performed 15 min before testing. The behaviour of the mice was recorded throughout the test, and the mice that held their hindlimbs or climbed their tails with their forelimbs during the tail suspension test were excluded from the analysis. An open field test was performed at least 2 days after the described tail suspension test^35^. An open field arena consisting of a white acrylic cube (50 × 50 × 50 cm) was used. Administration of each drug was performed 15 min before testing. The behaviour of each animal was recorded with a camera over a 10 min session; the recorded data were analysed automatically using a video tracking system (ANYmaze version 4.99, Stoelting, Wood Dale, IL, USA). The total distance travelled during each session was measured. All statistical tests were performed using Prism 8 (GraphPad Software). One-way ANOVA, followed by Dunnett’s multiple comparisons test, was used for group comparisons unless otherwise stated. The difference was considered significant at P < 0.05.

## Results and discussion

### Dataset

A benchmark dataset of 127 target proteins belonging to eight protein families was selected from ChEMBL by the procedure described in the previous section. Seven targets had a dataset size of fewer than 1000 (461–739), and 120 had a dataset size of more than 1000 (1408–11,632) (Supplementary Tables S2, S3).

The proper inclusion of inactive compounds has been shown to improve the prediction accuracy of classification models^6,36^. By analogy, it may be desirable for the dataset to have a wide range of activity values in the construction of regression models. Qualitative measurements above and below the detection limit of an assay, e.g., IC_50_ > 100,000 nM, were used “as is” without offsetting.

The distribution range of the reported pIC_50_ values directly influences R^2^, as shown in Eq. (3). The maximum and minimum pIC_50_ distribution ranges were 5.15 and 30.0 for the acetyl-CoA carboxylase 2 and alpha 1A adrenergic receptors, respectively. The large value of 30.0 was due to a compound of logK_i_ = 19, which might have been incorrectly registered in ChEMBL (the original paper listed it as 19% inhibition at 1 μM^37^). Although extreme outliers may negatively influence the predictability, we included them if the R^2^ value for a validation set was greater than the thresholds described in the previous section.

After the random splitting of the dataset, the validation sets were used to optimize the hyperparameters, and the test sets were used to evaluate the predictability of the models.

### Hyperparameter optimization and model training

Similar to other machine learning methods, a GCN is very sensitive to the choice of hyperparameters^38^. Table 1 shows the parameters searched and their explored ranges. The upper limit of the size of the graph convolutional layers is 9 to 32 times the value reported in the classification tasks^6–10^. For the parameters not listed in the table, the default values of DeepChem were used. Note that for small datasets, we limited the range to mitigate overfitting and underlearning problems.

**Table 1:** Hyperparameters and values explored.

Hyperparameter	Values explored	Values explored (for smaller datasets)
Size of the graph convolutional layers	[32–2048]	[16–512]
Size of the dense layer	[16–2048]	[16–512]
Number of graph convolutional layers	1, 2, 3–4	1, 2, 3–4
Learning rate	[0.00010–0.0020]	[0.00010–0.0020]
Dropout	[0.0–0.50]	[0.0–0.50]
Epoch	[20–200]	[20–200]
Batch size	[10–100]	[10–100]

R^2^, MAE, and RMSE values are often used to evaluate the performance of regression models. An R^2^ value of 1 indicates a perfect prediction, and a lower value indicates poor prediction accuracy, making it easier to intuitively judge the performance of a model. However, since R^2^ is affected by the activity range of the dataset used, as shown in Eq. (4), a careful comparison of performance is necessary between models of different datasets. Unlike R^2^, the lower the MAE or RMSE is, the better. There are some recommendations and concerns as to which metric should be used^39,40^.

The relationship between the two is described in Eq. (10).

10 $$\mathrm{MAE }\le \mathrm{ RMSE }\le \sqrt{n}\mathrm{MAE}$$

The upper bound of the RMSE is equal to the MAE multiplied by the square root of the dataset size $n$, which means that the RMSE tends to increase as the dataset size increases, implying that evaluating the model performance across different dataset sizes can be difficult. Furthermore, during the investigation of the MAE, RMSE, and R^2^ of the various parameter sets obtained by the hyperparameter search, we noticed that there were hyperparameter sets whose MAE values were only slightly worse than the smallest MAE value (e.g., 0.84 vs. 0.86) even if their R^2^ values were better (0.54 vs. 0.67). For these reasons, we evaluated the hyperparameter sets using the MAE and 2R2_MAE. R^2^ usually takes a value of [0–1]. MAE takes a value of [0–$\infty$], which differs from R^2^ in units. In our dataset, the MAE values are approximately in the range of [0–1], and we thought that it would not cause a significant problem to apply the arithmetical operations of the R^2^ and MAE as in Eq. (6) to perform a realistic assessment of the hyperparameter sets.

**Figure 1:**
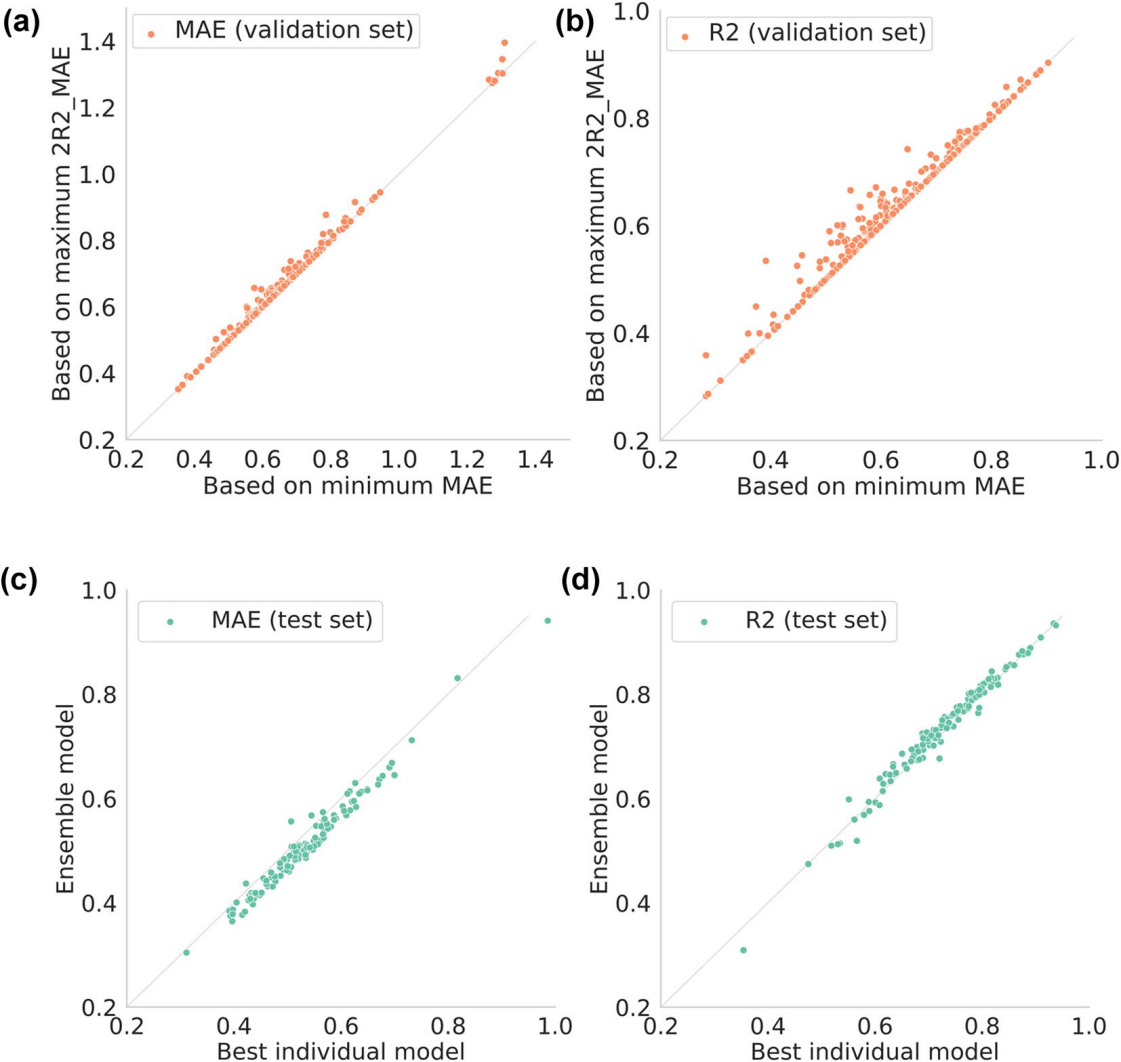
The impact of 2R2_MAE metric-based model selection and ensemble learning on the predictive performance. (**a**,**b**) Comparison of the MAE (**a**) and R^2^ (**b**) given by the hyperparameter sets selected according to the minimum MAE and maximum 2R2_MAE criteria. The points on the diagonal line represent cases in which the same hyperparameter set was selected by both criteria. There is no considerable difference in the MAE values under either criterion. The R^2^ values tend to improve when the hyperparameter set is selected by the maximum 2R2_MAE criterion. (**c**,**d**) Comparison of the MAE (**c**) and R^2^ (**d**) for the ensemble and best individual models. Ensemble learning resulted in a decrease in MAE values and a significant increase in R^2^ values.

Since 2R2_MAE is based on the balance between the R^2^ and the MAE, there is a concern that it may be the case that R^2^ is high (desirable), the MAE is high (undesirable), and 2R2_MAE is high (appears to be desirable). To investigate this problem, we comparatively analysed how the values of the MAE and R^2^ for the validation sets were affected by the hyperparameter combinations selected based on the criteria of the maximum 2R2_MAE and minimum MAE, respectively. As a result, for the hyperparameter sets selected with a maximum 2R2_MAE value, the MAE values were slightly worse than for those with a minimum MAE (the average increase was 0.0046; the maximum increase was 0.092), while the R^2^ values tended to be better (the average increase was 0.0082; the maximum increase was 0.14). Overall, the choice of hyperparameters based on the 2R2_MAE criterion seemed to provide reasonable models in our dataset (Fig. 1a,b).

The sizes of the convolutional layers and the dense layers varied with the dataset, and at the same time, their sizes tended to be close to the upper limit of the parameter search range. This result is similar to that in previous reports^12,23^, where activity classification models with large hidden layers showed good performance.

The training sets were retrained with a fixed random seed using the best hyperparameter sets (except for the epochs) that met the maximum 2R2_MAE criterion. Some models did not reproduce the prediction performance on the validation set within a reasonable range after retraining. For example, a hyperparameter set that showed R^2^ = 0.53 in the hyperparameter optimization process had R^2^ = 0.16 after the retraining. A lack of reproducibility was found in approximately 1.2% of the total models, but such models were excluded in ensemble learning, resulting in six to nine individual models per target.

In general, a DNN with more hidden layers better enables the extraction of complex high-level features and shows better performance. On the other hand, most of the models with a good performance in our study had one convolutional layer, and the models with four convolutional layers never outperformed those with three convolutional layers for any target during the hyperparameter search. One possible explanation for this apparent discrepancy is that the max-pooling layer not only extracts the features of a compound but also makes the information unnecessarily coarse. A GCN is essentially a type of Laplacian smoothing, and it has been pointed out that the repeated application of Laplacian smoothing may make the local chemical environment of compounds indistinguishable, which could explain our results^41^. To take advantage of the feature of graph convolution, in which the information of more distant atoms can be taken in as the layers increase, there is room left for improvement of the present architecture.

### Ensemble learning

The predictions made by individual models were averaged without weighting to generate ensemble predictions. Figure 1c,d compare the MAE and R^2^ on the test set. In Fig. 1c, the spots in the area below the diagonal line indicate a better performance in ensemble learning, and 120 targets fall in this area. In Fig. 1d, the spots above the diagonal line indicate that the ensemble predictions achieve better outcomes than the best individual models, and 94 targets are in this area. The statistical significance of the differences in the means of the MAE and R^2^ distributions between the best individual model and ensemble learning was tested with a one-sided Wilcoxon signed-rank test. The null hypothesis was rejected with P = 5.51 × 10^–20^ and 1.02 × 10^–8^, respectively, indicating that ensemble learning gave a lower mean MAE and a higher mean R^2^. The performance improvement with ensemble models is consistent with that obtained in other studies^23,27–29^. This improvement suggests that there can be many quasi-optimal hyperparameter combinations, and therefore, even similar combinations may capture different characteristics of compounds.

As a rule of thumb, we consider a model that satisfies either MAE < 0.6 or R^2^ > 0.6 to be a good model. In the present study, 86% (111 targets) and 91% (116 targets) of the models met the criteria of MAE < 0.6 and R^2^ > 0.6, respectively. Overall, the models quantitatively predicted the activity of a wide range of target proteins. The top four ensemble models based on the MAE values for each protein family and their corresponding individual models are presented in Table 2. The details of all targets are provided in Supplementary Table S3.

**Table 2:** The top four ensemble models for each protein family based on the MAE values (ensemble).

Protein family	Target	Size*	MAE: ensemble	R^2^: ensemble	MAE: individual model
GPCR	Orexin receptor 1	2852	0.36	0.79	0.41 ± 0.013
	Serotonin 7 (5-HT7) receptor	2395	0.42	0.74	0.47 ± 0.023
	Orexin receptor 2	3079	0.45	0.71	0.50 ± 0.010
	Cannabinoid CB1 receptor	6966	0.46	0.76	0.51 ± 0.0080
Enzyme	Acetyl-CoA carboxylase 2	3136	0.30	0.68	0.33 ± 0.018
	Poly [ADP-ribose] polymerase-1	3101	0.38	0.82	0.42 ± 0.012
	Cholinesterase	3011	0.39	0.82	0.43 ± 0.015
	Nicotinamide phosphoribosyltransferase	2342	0.41	0.68	0.45 ± 0.011
Ion channel	HERG	9198	0.38	0.66	0.42 ± 0.013
	Voltage-gated potassium channel subunit Kv1.5	739	0.39	0.53	0.42 ± 0.020
	Sodium channel protein type IX alpha subunit	5677	0.42	0.72	0.47 ± 0.016
	Vanilloid receptor	2856	0.46	0.78	0.50 ± 0.017
Kinase	Nerve growth factor receptor Trk-A	2587	0.37	0.71	0.42 ± 0.017
	Insulin-like growth factor I receptor	3019	0.40	0.85	0.44 ± 0.010
	Tyrosine-protein kinase JAK1	4345	0.41	0.81	0.45 ± 0.012
	Serine/threonine-protein kinase mTOR	4414	0.41	0.81	0.46 ± 0.018
Nuclear receptor	Thyroid hormone receptor alpha	461	0.38	0.82	0.40 ± 0.014
	Glucocorticoid receptor	2293	0.48	0.78	0.53 ± 0.026
	Peroxisome proliferator-activated receptor-gamma	3018	0.51	0.72	0.55 ± 0.015
	Vitamin D receptor	546	0.51	0.88	0.54 ± 0.030
Protease	Cathepsin D	2568	0.39	0.85	0.42 ± 0.018
	Matrix metalloproteinase-1	3746	0.42	0.81	0.47 ± 0.020
	ADAM17	2410	0.42	0.89	0.47 ± 0.022
	Cathepsin S	2309	0.46	0.79	0.50 ± 0.010
Trans-porter	Potassium-transporting ATPase	532	0.40	0.52	0.42 ± 0.0081
	GABA transporter 1	576	0.44	0.86	0.47 ± 0.040
	Dopamine transporter	5908	0.48	0.76	0.54 ± 0.014
	Norepinephrine transporter	4342	0.50	0.70	0.55 ± 0.015
Others	Histone deacetylase 1	4239	0.41	0.74	0.47 ± 0.015
	Bromodomain-containing protein 4	2208	0.41	0.82	0.46 ± 0.032
	Histone deacetylase 6	2725	0.42	0.82	0.47 ± 0.023
	p53-binding protein Mdm-2	2346	0.42	0.88	0.47 ± 0.020

*Size: The number of compounds in the dataset.

**Figure 2:**
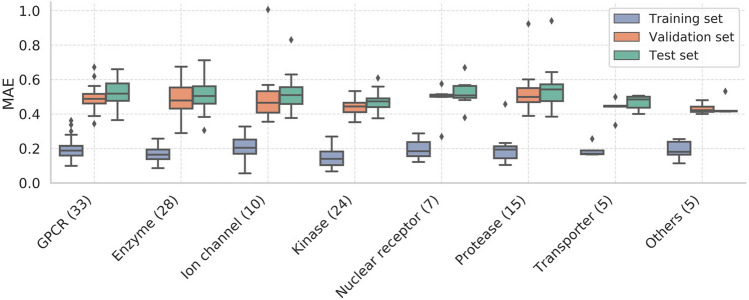
Box-whisker plots of the MAE of the ensemble models for each protein family. The horizontal lines in the boxes indicate the medians, the ends of the whiskers indicate the maximum and minimum MAE values, the bottoms and tops of the boxes are the 25th and 75th percentiles, and the points outside the whiskers are outliers. The number after each name on the x-axis shows the number of targets in each family. The same colour code for the data subsets is used throughout this manuscript.

Figure 2 shows the performance of ensemble learning for each of eight protein families. The MAE at the 75th percentile (third quartile) of all protein families was less than 0.6. Only two targets exceeded 0.8, i.e., neuronal acetylcholine receptor alpha4/beta2 and human immunodeficiency virus type 1 protease, probably because approximately 2% of the compounds consistently showed remarkably low predicted pIC_50_ values, which increased the MAE. The MAE values for the validation and test sets tended to be larger than those of the training sets, suggesting that some degree of overlearning occurred, although most of the MAE values met our criterion of MAE < 0.6.

### Comparison with convolutional neural network models on image data (KekuleScope)

A convolution operation on a two-dimensional image of a compound has been used for the qualitative and quantitative prediction of toxicity and pharmacological activity. The input image can be either a two-dimensional sketch^42,43^ or a snapshot of a compound drawn as a three-dimensional picture^44^. The feature vector of each compound is extracted by a convolutional operation on its image data^42–45^.

Cortés-Ciriano et al. studied two-dimensional compound image data with architectures widely used in image recognition, ResNet-52 and VGG-19, and reported that their models (KekuleScope) quantitatively predicted pIC_50_ for 25 target proteins from ChEMBL (release 23)^43^. We built GCN models for the KekuleScope dataset and compared the RMSE values with those of the KekuleScope. As a result, our RMSE values were equivalent to those of the KekuleScope and, although indirectly, were close to those of the random forest (RF) models and fully connected deep neural network (FNN) models reported simultaneously (Table 3, Supplementary Table S4). In addition, we compared the RMSE values of our 22 models built using ChEBML (release 25) and found that all values were equivalent to or lower than those of the KekuleScope, RF, and FNN. This observation suggests that sufficient features can be extracted from the two-dimensional structures to predict their activity.

**Table 3:** Comparison with the KekuleScope.

	Model	RMSE^a^	RMSE^b^
KekuleScope	CNN	0.76 ± 0.078	
	RF	0.68 ± 0.070	
	FNN	0.71 ± 0.076	
Present study	GCN	0.74 ± 0.091	0.49 ± 0.11

^a^The KekuleScope dataset.

^b^ChEMBL (release 25).

### Impact of the scaffold diversity and dataset size

The diversity of structures in datasets, especially training data, should be considered within the context of the applicability domain of a model. A widely accepted definition of structural diversity is in terms of Murcko’s scaffolds^30^. Many reports have applied these scaffolds to evaluate the structural diversity of datasets^23,46,47^ and have generated compounds with privileged scaffolds for the expression of the activity of interest^48^. There were 145,515 unique scaffolds in ChEMBL, from the insulin-like growth factor I receptor (707 scaffolds) to carbonic anhydrase XII (356 scaffolds).

**Figure 3:**
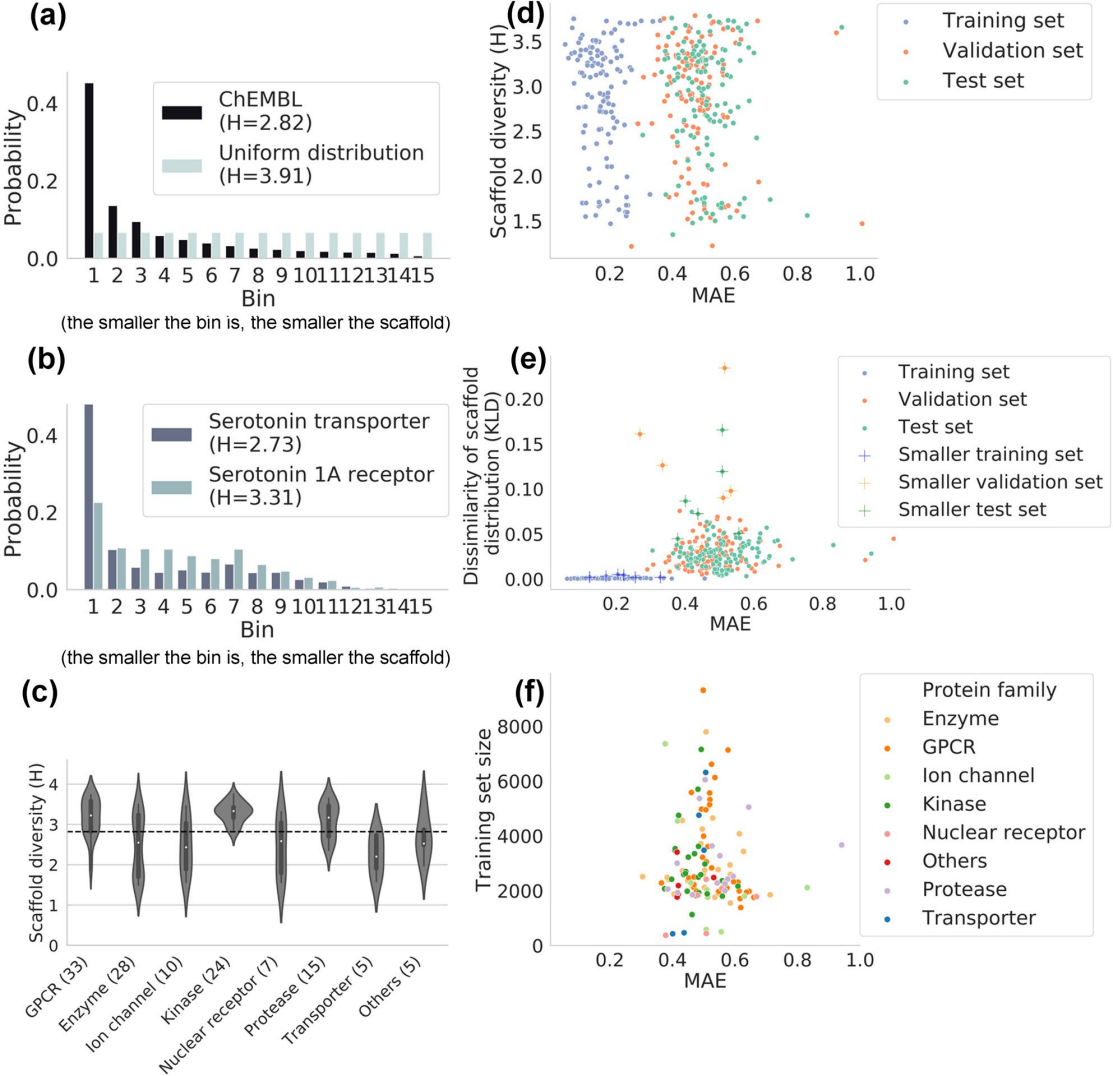
Effect of the scaffold diversity of the datasets on the prediction performance. (**a**) The probability distribution of the scaffolds in ChEMBL. Compared to the uniform distribution, ChEMBL is much more biased towards smaller scaffolds, resulting in a smaller H value. (**b**) The probability distribution of the scaffolds in the dataset for the serotonin transporter (SERT) and serotonin 1A receptor (5-HT1A). The H value is larger for 5-HT1A, whose scaffold distribution is wider than that of SERT. (**c**) Violin plots of the H value distribution by protein family. The number after each name on the x-axis shows the number of targets in each family. (**d**–**f**) Effect of the scaffold diversity (**d**), the dissimilarity of the scaffold distribution (**e**), and the training set size (**f**) on the MAE.

Even if targets A and B contain the same scaffolds, whether the distribution of the scaffolds is equal is another question. Target A may consist mostly of compounds with small scaffolds, while most of the compounds in target B may have large scaffolds. To analyse the relationship between structural diversity and the scaffold distribution, we applied the Shannon entropy (H) as a scaffold diversity measure, which can quantitatively convert various continuous and discontinuous data distributions into their information content (Eq. (7)). When the scaffold distribution is represented by a histogram, the H value is independent of the size of the bin interval if it is divided into the same number of bins and the same range. In a 15-bin histogram, as we used in our dataset, H takes values from zero to 3.91. ChEMBL itself is 2.82, meaning that it deviates from a uniform distribution (H = 3.91). When considered in conjunction with the probability distribution, we find that the deviation is associated with a bias towards smaller scaffolds (Fig. 3a). SERT has a similar probability distribution as ChEMBL and a similar value of H (2.73), while serotonin 1A receptor (5-HT1A), which, like SERT, recognizes serotonin, shows an even distribution from the second bin to the seventh, with an H value of 3.31 (Fig. 3b). Details of all targets are given in Supplementary Table S3.

The violin plots in Fig. 3c depict the distribution of H values for each protein family. The horizontal dashed line indicates 2.82 (the H value of ChEMBL). The first quartiles of the H value distributions for GPCRs and kinases are greater than 2.82, indicating that many targets have a higher scaffold diversity. Enzymes, ion channels, and nuclear receptors exhibit a wide range of targets, from scaffolds with a high diversity to a limited diversity.

The importance of selecting various chemical compounds in the initial screening has been consistently reported^49,50^. The greater the structural diversity of a training set is and the more scaffolds there are, the larger the applicability domain of the model^6^. From this point of view, we evaluated the relationship between H and MAE for our dataset. As shown in Fig. 3d, no correlation was observed. The Spearman rank-order correlation coefficients were − 0.11, − 0.023, and − 0.062 for the training, validation, and test sets, respectively. These results indicate that the resulting models are generally predictive for any dataset regardless of the diversity of the scaffolds.

The differences in the distribution of scaffolds between split datasets can affect the performance of models since the adoption of a non-scaffold-overlapping approach has been reported to tend to reduce the predictability of models^10,28^. Even if random splitting is applied, an uneven scaffold distribution between datasets could unintentionally occur, especially for smaller datasets. Therefore, we introduced the KLD (Eq. (9)) to quantify the differences in the scaffold distribution between datasets. When the scaffold distribution is the same between the split datasets compared, the KLD has a minimum value of zero. Even if two split datasets produce the same H value, they do not necessarily have the same scaffold distribution, and the greater the difference in the distribution is, the greater the KLD value.

Figure 3e illustrates the relationship between the KLD and MAE. A plus sign means a small dataset of fewer than 1000 compounds. As expected, the training sets (blue dots) have very small KLD values for all targets, which explains the nearly identical scaffold distribution before and after the split. Most of the KLD values of the validation and test sets split from more than 1000 compounds show similar KLD distributions below 0.07, suggesting that the random split functions are as expected. For most of the small datasets, the KLD values are larger than 0.07, indicating that the scaffold distribution was unintentionally biased. For the 127 targets we studied, there was no correlation between the KLD and MAE. The Spearman rank-order correlation coefficients are 0.094, 0.16, and 0.068 for the training, validation, and test sets, respectively. Moreover, even for targets with small dataset sizes, the MAE ranges from 0.1 to 0.6, despite the large KLD values. These results suggest that a difference in scaffold distributions within this range does not have a clear impact on the model performance.

Figure 3f compares the size of the training set with the MAE of the test set. Again, there is no clear correlation between the dataset size and the MAE. There is also no apparent tendency to favour specific protein families. However, as pointed out in another report^51^, in small datasets, it may be less sensible to assess the performance of the models at face value due to inherent problems such as over- and under-learning and the relative noise impact.

### Impact of the data splitting

Two targets with the largest and two targets with the smallest datasets were selected for each protein family to investigate the effect of data splitting on model performance. For each target, we repeated the random split of the training-validation set twice to generate three datasets in total (SET1, 2, and 3) (Supplementary Fig. S2). The GCN models built for these three datasets showed equivalent predictive performance (Supplementary Table S5).

### Virtual screening

**Figure 4:**
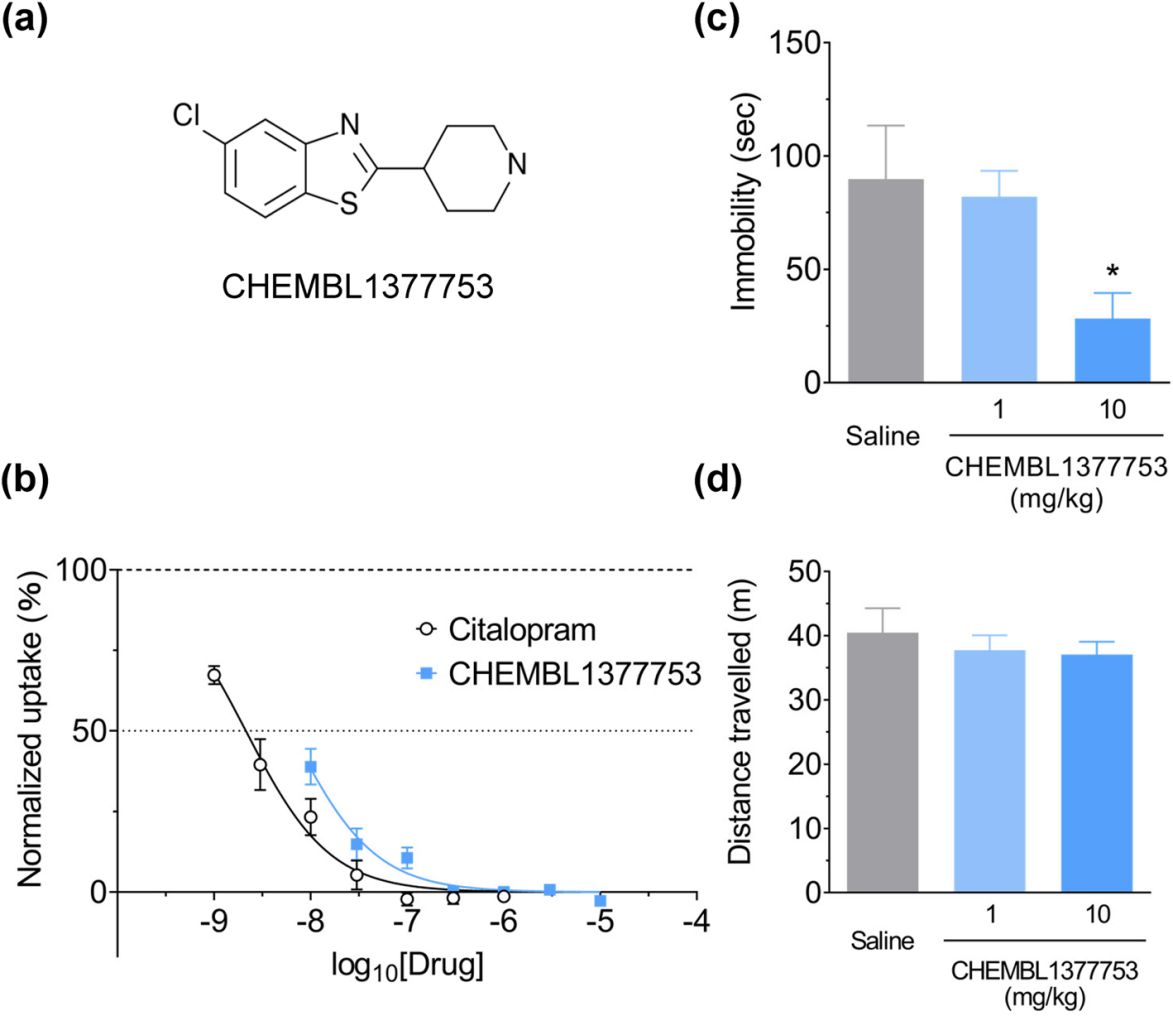
Experimental validation of the prediction model for the serotonin transporter (SERT). (**a**) Structure of CHEMBL1377753 (**1**). (**b**) **1** inhibited the substrate uptake of SERT. The specific uptake of the fluorescent substrate for SERT was measured in the absence or presence of ligands in cells expressing human SERT. The specific uptake was normalized to the value in the absence of ligands. The data represent the mean ± s.e.m. n = 4 biological replicates in two independent experiments. (**c**,**d**) Administration of **1** induced antidepressant-like effects in mice. After intraperitoneal injection of **1** (1, 10 mg/kg), the immobility duration in the tail suspension test (**c**) or travelled distance in the open field test (**d**) was measured. (**c**) **1** significantly decreased the immobility duration. The data represent the mean ± s.e.m. One-way ANOVA was performed; F(2,19) = 3.64, P = 0.046. Dunnett’s multiple comparisons test *P < 0.05 vs. the saline group. n = 7–8 mice per group. (**d**) **1** did not significantly affect the travelled distance. The data represent the mean ± s.e.m. One-way ANOVA; F(2,15) = 0.41, P = 0.67. n = 6 mice per group.

To further evaluate the performance of our models, the SERT activity was calculated for 1,777,353 compounds from ChEMBL processed as described in the Materials and Methods section, except for the assay_type = B filter. Since the octanol/water distribution coefficient (logP) values of the marketed selective serotonin reuptake inhibitors (SSRIs) are in the range of 2.29 to 5.15 (calculated with IJC), the compounds were narrowed down using a logP filter. From the compounds that satisfied logP > 2, a predicted pIC_50_ ≥ 7.5 for SERT, a pIC_50_ ≤ 6.0 for the 5-HT1A receptor, and no assay reports for monoamine-related proteins or opioid receptors (SERT, the other serotonin receptors, dopamine receptors and transporter, opioid receptors, and adrenergic receptors), after visual inspection, a readily available **1** (Fig. 4a) was purchased and subjected to a pharmacological activity test.

### In vitro assays

We measured the inhibition activity of **1,** whose predicted IC_50_ was approximately 10 nM (pIC_50_ = 7.97), in HEK293 cells transiently expressing SERT. Specific uptake by SERT was inhibited by **1** as well as citalopram, an SSRI, in a dose-dependent manner (Fig. 4b). Non-linear regression analyses revealed that the IC_50_ values of **1** and citalopram were 6.24 nM and 2.13 nM, respectively.

When the structural similarity to **1** was calculated through IJC on 10,270 ChEMBL compounds with activity data for SERT, the reported pIC_50_ for the most similar compound (Tanimoto coefficient = 0.78; the larger the value is, the more similar the compounds) was 5.93, which was 100 times weaker than the activity of **1**. Additionally, the similarity of **1** to the most active compound (reported pIC_50_ = 11.70; a calcilytic agent that had been investigated as a calcium-sensing receptor antagonist) was 0.26. It is often advised that compounds sharing the same scaffold should not be used simultaneously in the training, validation, and test sets. However, our results suggest that GCN models learn the relationship between the local chemical environments and the activity values of compounds and that less control over the scaffold distribution may be required.

### Behavioural tests

Because SSRIs are widely used as antidepressants^52^, we investigated whether **1** had antidepressant-like efficacy in mice. Administration of **1** (10 mg/kg, i.p.) significantly reduced the immobility duration in the tail suspension test, a proxy of a depression-like state, whereas it did not affect general locomotor activity in the open field test (Fig. 4c,d). Judging from the logP value of 2.94, it is possible that **1** was distributed in the central system and may have shown antidepressant effects. In ChEMBL, activity of **1** against transient receptor potential canonical 4 (pIC_50_ = 8.10) and nuclear factor erythroid 2-related factor 2 (pIC_50_ = 6.19) has been reported. Thus, it is also possible to assume that the antidepressant effects occurred by acting on these two or other unknown targets.

## Conclusions

Quantitative activity prediction models were constructed for 127 target proteins in ChEMBL using only features extracted from the two-dimensional structural information of compounds by applying a GCN architecture. We extended the range of hyperparameters beyond the range reported in the classification tasks. Most of the models with good performance in this study had one convolutional layer, and none of the models with four convolutional layers outperformed the three-layer models during the hyperparameter search. Ensemble learning improved the predictive performance compared to the individual models.

The prediction performance of GCN models built using the KekuleScope dataset was comparable to that of the KekuleScope (CNN) model and, indirectly, RF and FNN models which are often used for comparison purposes as baseline methods. Interestingly, our models built using ChEMBL (release 25) showed a better performance than the CNN, RF, and FNN models, although it should be noted that the data preparation scheme and handling of qualitative measurements in the KekuleScope dataset differ from those in our dataset.

Databases collected from various data sources contain measurements and noise under various experimental conditions such as a template and a substrate for reverse transcriptase^53^. By taking these factors into account, the performance of activity prediction models has been improved^53^. Since only the filters described in the Materials and Methods section were used in this study (the standard relation was one of “>”, “≥”, “=”, “≤”, and “<”; excluded inconclusive data, duplicates, and errors), further investigation is needed. For instance, when multiple experimental values were available for the same compound-target pair, the maximum value was used in the present study as previously reported^6,9,18^, but other lines of reports used the median^11,23,53^ and the mean^43^ values, thus a different data pre-processing method may further improve the prediction performance of the models.

In addition to constructing the models, we quantified the diversity of the compound scaffolds and demonstrated that the diversity had less effect on the model performance. The virtual screening performed to further validate the generalizability of our models identified a new compound with SERT activity, which is comparable to citalopram.

Even if the targets on which activity prediction models are built are “unappealing”, the models can provide useful hints for drug repositioning, alerting to potential off-targets, prioritizing strategies in the early stage of drug development, finding poly-pharmacological drugs, and searching for tool compounds that support the elucidation of the molecular mechanisms underlying biological function. From this point of view, we believe that a model that ranks compounds not by binary classification but by quantitative prediction is a useful tool in drug discovery research. We believe that our GCN architecture could play a crucial part in such an effort, as we obtained a novel SERT-acting compound with activity comparable to that of a clinically effective drug.

## Supplementary Information

Supplementary Information.

## Data Availability

The codes and datasets used in this study are available from the corresponding author on request.
