# Supplementary material for: Prediction of pharmacological activities from chemical structures with graph convolutional neural networks
Source: Sci Rep. 2021 Jan 12;11:525. doi: 10.1038/s41598-020-80113-7 (PMC7803991; doi:10.1038/s41598-020-80113-7)
Supplement: Supplementary file 1 — Supplementary Information. [file 41598_2020_80113_MOESM1_ESM.pdf]

Supplementary Information

Prediction of pharmacological activities from chemical structures with graph convolutional neural networks

Miyuki Sakai, Kazuki Nagayasu, Norihiro Shibui, Chihiro Andoh, Kaito Takayama, Hisashi Shirakawa and Shuji Kaneko

Supplementary Figure S1, S2
Supplementary Table S1-S5

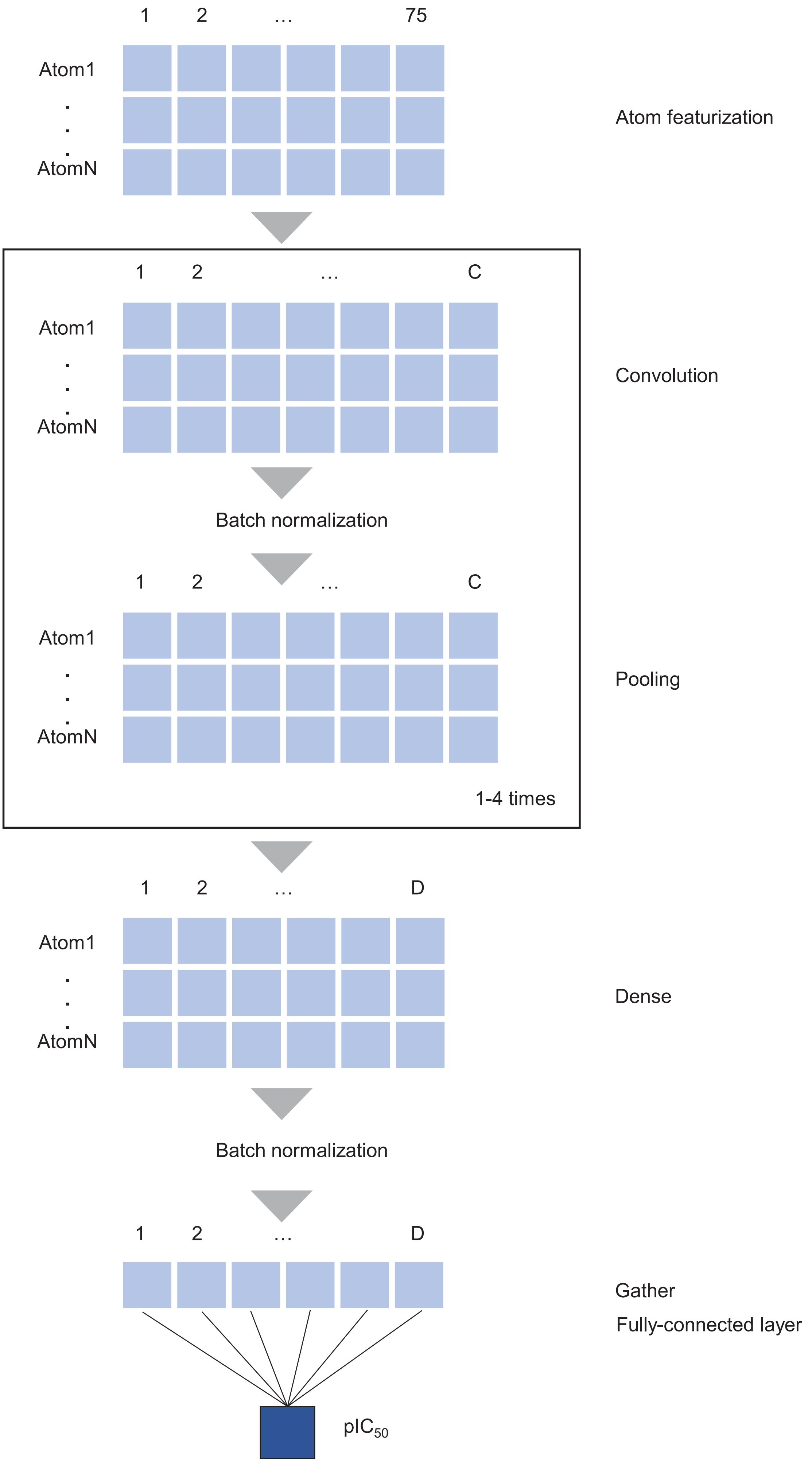

Figure S1. Schematic of graph convolutional neural network architecture

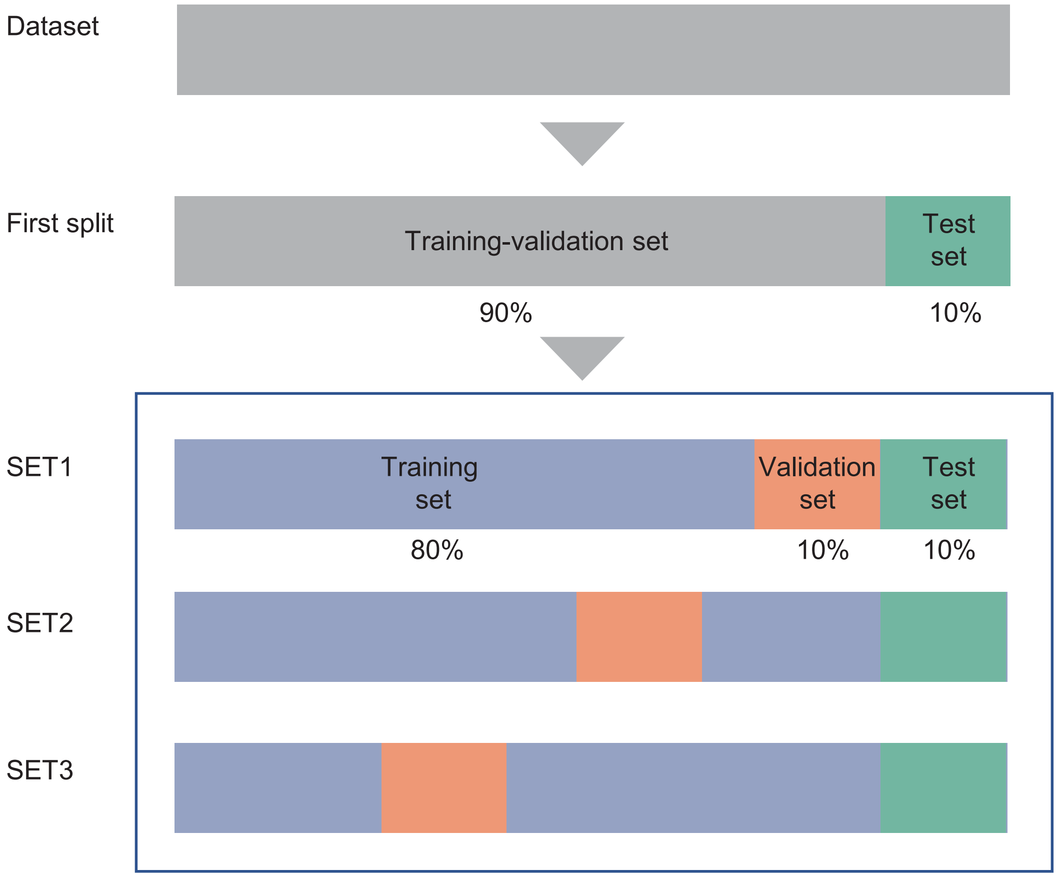

Figure S2. Data splitting

Table S1. Atom features

Feature	Description	Size
atom type	type of atom (e.g., C, N, O, S) and "Unknown" (one-hot)	44
degree	number of bonds the atom is connected to (one-hot)	11
valence	number of implicit valence (one-hot)	7
formal charge	integer electronic charge	1
radical electrons	number of radical electrons	1
hybridization	sp, sp2, sp3, sp3d, or sp3d2 (one-hot)	5
aromaticity	whether the atom is part of an aromatic system	1
hydrogens	number of bonded hydrogen atoms (one-hot)	5

Table S2. Protein families and targets

Protein family	Number of targets[a]	Number of targets[b]
G protein-coupled receptor (GPCR)	33	0
Enzyme[c]	28	0
Kinase	24	0
Protease	15	0
Ion channel	7	3
Nuclear receptor	5	2
Transporter	3	2
Others	5	0

a: The number of targets containing compounds equal to or greater than 1,000

b: The number of targets containing compounds fewer than 1,000

c: In the classification of ChEMBL, “kinase” and “protease” belong to the subcategory of “enzyme”, however, these two classes are counted separately, and "enzyme" in this report does not include "kinase" nor "protease".

Table S3. All targets studied and their details

	Protein family	Target[a]	Number of compounds	Scaffold diversity (H)	Number of convolutional layers	Size: convolutional layer	Size: dense layer	MAE: ensemble	R^2: ensemble	MAE: individual model[c]	R^2: individual model[c]
1	GPCR	Orexin receptor 1	2,852	3.13	1-2	52 - 1,489	688 - 2,028	0.36	0.79	0.41 ± 0.013	0.77 ± 0.013
2	GPCR	Serotonin 7 (5-HT7) receptor	2,395	3.36	1-2	99 - 1,978	420 - 2,031	0.42	0.74	0.47 ± 0.023	0.70 ± 0.026
3	GPCR	Orexin receptor 2	3,079	3.11	1-2	248 - 1,708	621 - 2,026	0.45	0.71	0.50 ± 0.010	0.68 ± 0.015
4	GPCR	Cannabinoid CB1 receptor	6,966	2.90	1-2	99 - 1,460	979 - 2,031	0.46	0.76	0.51 ± 0.0080	0.72 ± 0.0076
5	GPCR	G protein-coupled receptor 44	2,655	2.52	1-2	64 - 1,238	740 - 1,889	0.47	0.65	0.52 ± 0.037	0.59 ± 0.040
6	GPCR	Dopamine D1 receptor	2,774	2.82	1-2	232 - 1,014	999 - 2,041	0.47	0.71	0.52 ± 0.021	0.69 ± 0.013
7	GPCR	Melanin-concentrating hormone receptor 1	3,124	3.48	1-2	97 - 1,909	896 - 2,038	0.47	0.60	0.52 ± 0.0078	0.54 ± 0.011
8	GPCR	Serotonin 6 (5-HT6) receptor	3,106	2.78	1-2	50 - 2,027	227 - 2,045	0.47	0.72	0.53 ± 0.018	0.67 ± 0.019
9	GPCR	Corticotropin releasing factor receptor 1	2,471	1.87	1-2	295 - 1,561	829 - 1,998	0.48	0.63	0.52 ± 0.020	0.59 ± 0.025
10	GPCR	Adenosine A2a receptor	6,206	2.91	1-2	317 - 1,862	788 - 1,974	0.49	0.69	0.55 ± 0.019	0.64 ± 0.023
11	GPCR	Histamine H3 receptor	4,021	2.88	1-2	114 - 1,245	964 - 1,977	0.50	0.65	0.55 ± 0.012	0.60 ± 0.016
12	GPCR	Dopamine D2 receptor	11,632	3.35	1-2	129 - 1,148	1,038 - 1,929	0.50	0.67	0.55 ± 0.0082	0.62 ± 0.0085
13	GPCR	Dopamine D3 receptor	4,983	3.45	1-2	206 - 1,982	1,297 - 1,943	0.50	0.72	0.55 ± 0.018	0.68 ± 0.019
14	GPCR	Serotonin 2a (5-HT2a) receptor	6,189	3.25	1-2	87 - 1,758	882 - 1,992	0.51	0.69	0.56 ± 0.010	0.65 ± 0.015
15	GPCR	Serotonin 2c (5-HT2c) receptor	3,807	2.81	1-2	91 - 1,703	558 - 1,994	0.51	0.72	0.57 ± 0.020	0.67 ± 0.011
16	GPCR	Adenosine A3 receptor	4,502	3.00	1-2	99 - 1,279	757 - 2,031	0.51	0.72	0.57 ± 0.018	0.68 ± 0.013
17	GPCR	Delta opioid receptor	6,946	3.74	1-2	157 - 1,764	411 - 1,911	0.52	0.78	0.58 ± 0.019	0.73 ± 0.019
18	GPCR	Cannabinoid CB2 receptor	6,413	2.71	1-2	150 - 1,577	371 - 1,980	0.52	0.70	0.59 ± 0.022	0.65 ± 0.022
19	GPCR	Kappa opioid receptor	6,645	3.60	1-2	211 - 2,044	1,166 - 1,953	0.52	0.75	0.59 ± 0.031	0.70 ± 0.027
20	GPCR	Serotonin 1a (5-HT1a) receptor	8,261	3.31	1-2	97 - 1,860	697 - 2,021	0.52	0.69	0.58 ± 0.012	0.64 ± 0.0086
21	GPCR	Metabotropic glutamate receptor 5	3,535	2.10	1-2	175 - 1,604	637 - 1,827	0.53	0.67	0.57 ± 0.016	0.66 ± 0.020
22	GPCR	Adenosine A1 receptor	7,650	2.78	1-2	291 - 2,036	1,365 - 2,028	0.54	0.65	0.59 ± 0.019	0.60 ± 0.022
23	GPCR	Cholecystokinin B receptor	2,459	3.74	1-2	163 - 1,253	722 - 1,994	0.55	0.75	0.59 ± 0.021	0.73 ± 0.016
24	GPCR	Dopamine D4 receptor	2,622	3.11	1-2	125 - 1,999	1,414 - 1,980	0.57	0.51	0.61 ± 0.027	0.48 ± 0.030
25	GPCR	Mu opioid receptor	8,904	3.71	1-2	75 - 1,282	897 - 1,808	0.58	0.73	0.63 ± 0.012	0.70 ± 0.011
26	GPCR	Melanocortin receptor 4	2,902	3.72	1-2	107 - 1,917	251 - 1,932	0.60	0.61	0.64 ± 0.026	0.59 ± 0.019
27	GPCR	Neurokinin 1 receptor	2,888	3.71	1-2	45 - 963	919 - 2,010	0.61	0.70	0.65 ± 0.020	0.68 ± 0.016
28	GPCR	Endothelin receptor ET-A	2,486	3.58	1-2	274 - 1,979	572 - 1,928	0.61	0.77	0.66 ± 0.022	0.74 ± 0.018
29	GPCR	Muscarinic acetylcholine receptor M2	2,135	3.22	1-2	45 - 1,457	588 - 1,927	0.62	0.83	0.69 ± 0.076	0.80 ± 0.037
30	GPCR	Gonadotropin-releasing hormone receptor	1,726	3.72	1-2	256 - 1,486	1,065 - 2,006	0.62	0.94	0.71 ± 0.052	0.91 ± 0.018
31	GPCR	Cholecystokinin A receptor	2,423	3.70	1-2	50 - 1,719	583 - 1,905	0.64	0.51	0.68 ± 0.024	0.50 ± 0.018
32	GPCR	Muscarinic acetylcholine receptor M1	2,465	2.78	1-2	88 - 1,308	562 - 2,014	0.65	0.80	0.71 ± 0.021	0.77 ± 0.030
33	GPCR	Alpha-1a adrenergic receptor	2,193	3.43	1-2	108 - 1,061	562 - 2,002	0.66	0.67	0.73 ± 0.023	0.64 ± 0.047
34	Enzyme	Acetyl-CoA carboxylase 2	3,136	2.56	1-2	99 - 1,867	849 - 2,035	0.30	0.68	0.33 ± 0.018	0.64 ± 0.032
35	Enzyme	Poly [ADP-ribose] polymerase-1	3,101	2.91	1-2	165 - 1,206	679 - 2,001	0.38	0.82	0.42 ± 0.010	0.79 ± 0.011
36	Enzyme	Cholinesterase	3,011	3.50	1-2	222 - 1,843	920 - 2,022	0.39	0.82	0.43 ± 0.015	0.79 ± 0.010
37	Enzyme	Nicotinamide phosphoribosyltransferase	2,342	3.12	1-2	166 - 1,592	679 - 1,945	0.41	0.68	0.45 ± 0.011	0.63 ± 0.023
38	Enzyme	PI3-kinase p110-alpha subunit	5,699	3.38	1-2	161 - 1,725	724 - 1,968	0.43	0.78	0.48 ± 0.012	0.75 ± 0.0074
39	Enzyme	11-beta-hydroxysteroid dehydrogenase 1	3,410	2.54	1-2	154 - 1,067	862 - 1,917	0.43	0.79	0.48 ± 0.0076	0.77 ± 0.0068
40	Enzyme	Arachidonate 5-lipoxygenase	2,953	2.09	1-2	94 - 1,574	649 - 1,837	0.45	0.64	0.49 ± 0.012	0.59 ± 0.016
41	Enzyme	Carbonic anhydrase XII	2,664	1.50	1-2	120 - 1,436	817 - 1,904	0.46	0.69	0.52 ± 0.023	0.65 ± 0.033
42	Enzyme	Phosphodiesterase 10A	4,506	3.29	1-2	231 - 1,502	854 - 2,033	0.49	0.75	0.55 ± 0.038	0.69 ± 0.030
43	Enzyme	Cyclooxygenase-1	4,635	1.69	1-2	58 - 1,330	553 - 1,897	0.49	0.47	0.54 ± 0.023	0.45 ± 0.028
44	Enzyme	Protein-tyrosine phosphatase 1B	3,263	3.04	1-2	264 - 1,073	577 - 1,979	0.50	0.59	0.55 ± 0.037	0.55 ± 0.039
45	Enzyme	Carbonic anhydrase I	5,305	1.71	1-2	366 - 1,904	1,150 - 1,814	0.50	0.74	0.55 ± 0.018	0.70 ± 0.013
46	Enzyme	PI3-kinase p110-delta subunit	2,647	3.31	1-2	46 - 1,365	640 - 1,991	0.50	0.71	0.56 ± 0.016	0.69 ± 0.010
47	Enzyme	Carbonic anhydrase IX	3,431	1.59	1-2	119 - 1,642	781 - 1,966	0.50	0.70	0.55 ± 0.018	0.67 ± 0.017
48	Enzyme	Acetylcholinesterase	9,737	3.26	1-2	103 - 1,299	687 - 2,044	0.51	0.71	0.57 ± 0.019	0.68 ± 0.011
49	Enzyme	Integrase	2,545	2.37	1-2	177 - 1,833	756 - 1,742	0.51	0.82	0.57 ± 0.032	0.79 ± 0.014
50	Enzyme	Carbonic anhydrase II	5,808	1.65	1-2	43 - 1,735	798 - 1,913	0.53	0.76	0.62 ± 0.030	0.71 ± 0.024
51	Enzyme	Cytochrome P450 19A1	2,196	1.62	1-2	272 - 1,768	681 - 1,967	0.54	0.73	0.58 ± 0.012	0.70 ± 0.011
52	Enzyme	Butyrylcholinesterase	3,451	3.25	1-2	67 - 1,645	938 - 2,014	0.55	0.73	0.61 ± 0.023	0.67 ± 0.039
53	Enzyme	Human immunodeficiency virus type 1 reverse transcriptase	2,310	1.70	1-2	179 - 1,433	825 - 1,968	0.55	0.68	0.59 ± 0.018	0.64 ± 0.022
54	Enzyme	Protein farnesyltransferase	2,653	3.34	1-2	190 - 1,638	333 - 1,947	0.56	0.77	0.64 ± 0.033	0.72 ± 0.025
55	Enzyme	PI3-kinase p110-gamma subunit	2,596	3.23	1-2	165 - 2,019	556 - 1,959	0.56	0.59	0.61 ± 0.019	0.57 ± 0.022
56	Enzyme	Monoamine oxidase A	3,666	1.61	1-2	269 - 1,281	741 - 1,985	0.58	0.63	0.65 ± 0.016	0.59 ± 0.019
57	Enzyme	Gamma-secretase	1,932	3.16	1-2	32 - 1,634	1,130 - 1,980	0.59	0.67	0.62 ± 0.017	0.63 ± 0.026
58	Enzyme	Anandamide amidohydrolase	3,242	2.72	1-2	254 - 1,871	1,339 - 2,005	0.59	0.86	0.66 ± 0.025	0.83 ± 0.014
59	Enzyme	Cyclooxygenase-2	5,085	1.72	1-2	72 - 1,441	562 - 1,915	0.61	0.68	0.68 ± 0.031	0.64 ± 0.023
60	Enzyme	Monoamine oxidase B	4,283	1.62	1-2	73 - 1,996	721 - 2,043	0.63	0.66	0.70 ± 0.023	0.63 ± 0.019
61	Enzyme	Dihydrofolate reductase	2,309 [b]	1.74	1-2	103 - 1,721	1,035 - 1,998	0.71	0.57	0.76 ± 0.017	0.57 ± 0.018
62	Ion channel	HERG	9,198	3.46	1-2	156 - 1,455	1,038 - 1,888	0.38	0.66	0.42 ± 0.013	0.62 ± 0.0087
63	Ion channel	Voltage-gated potassium channel subunit Kv1.5	739	3.27	1-2	94 - 430	151 - 495	0.39	0.53	0.42 ± 0.020	0.50 ± 0.029
64	Ion channel	Sodium channel protein type IX alpha subunit	5,677	3.13	1-2	125 - 1,331	887 - 1,907	0.42	0.72	0.47 ± 0.016	0.68 ± 0.020
65	Ion channel	Vanilloid receptor	2,856	2.56	1-2	45 - 1,898	342 - 1,728	0.46	0.78	0.50 ± 0.017	0.75 ± 0.016
66	Ion channel	Transient receptor potential cation channel subfamily M member 8	721	2.74	1-3	160 - 510	67 - 489	0.51	0.76	0.58 ± 0.040	0.73 ± 0.036
67	Ion channel	Apoptosis regulator Bcl-2	2,542	2.31	1-2	156 - 1,437	1,177 - 2,001	0.51	0.88	0.55 ± 0.026	0.86 ± 0.016
68	Ion channel	Neuronal acetylcholine receptor protein alpha-7 subunit	2,195	2.12	1-2	114 - 1,796	989 - 1,984	0.51	0.75	0.57 ± 0.014	0.72 ± 0.013
69	Ion channel	Transient receptor potential cation channel subfamily A member 1	621	1.81	1-2	41 - 399	269 - 467	0.56	0.68	0.57 ± 0.031	0.66 ± 0.035
70	Ion channel	P2X purinoceptor 7	2,247	1.79	1-2	68 - 664	591 - 1,719	0.63	0.31	0.65 ± 0.018	0.30 ± 0.031
71	Ion channel	Neuronal acetylcholine receptor; alpha4/beta2	2,625	1.56	1-2	36 - 1,926	690 - 2,000	0.83	0.51	0.90 ± 0.047	0.51 ± 0.025
72	Kinase	Nerve growth factor receptor Trk-A	2,587	3.36	1-2	196 - 1,694	856 - 1,938	0.37	0.71	0.42 ± 0.017	0.67 ± 0.025
73	Kinase	Insulin-like growth factor I receptor	3,019	3.76	1-2	98 - 1,731	495 - 1,642	0.40	0.85	0.44 ± 0.010	0.83 ± 0.0083
74	Kinase	Tyrosine-protein kinase JAK1	4,345	3.19	1-2	233 - 1,827	934 - 1,989	0.41	0.81	0.45 ± 0.012	0.78 ± 0.0093
75	Kinase	Serine/threonine-protein kinase mTOR	4,414	3.60	1-2	420 - 1,710	652 - 1,838	0.41	0.81	0.46 ± 0.018	0.78 ± 0.022
76	Kinase	Tyrosine-protein kinase JAK2	5,933	3.29	1-2	158 - 1,676	1,132 - 1,963	0.42	0.80	0.46 ± 0.010	0.77 ± 0.0072
77	Kinase	Serine/threonine-protein kinase B-raf	3,234	3.54	1-2	68 - 1,500	586 - 1,994	0.44	0.80	0.48 ± 0.012	0.77 ± 0.011
78	Kinase	Hepatocyte growth factor receptor	3,276	3.66	1-2	126 - 2,011	687 - 1,954	0.44	0.80	0.51 ± 0.032	0.75 ± 0.037
79	Kinase	Tyrosine-protein kinase SYK	3,368	3.39	1-2	52 - 1,737	1,146 - 1,939	0.44	0.80	0.49 ± 0.015	0.78 ± 0.0057
80	Kinase	Fibroblast growth factor receptor 1	2,473	3.31	1-2	87 - 1,726	572 - 1,909	0.45	0.83	0.49 ± 0.024	0.81 ± 0.017
81	Kinase	Serine/threonine-protein kinase PIM1	4,022	2.93	1-2	193 - 1,494	1,144 - 1,996	0.45	0.88	0.49 ± 0.012	0.86 ± 0.0070
82	Kinase	Fibroblast growth factor receptor 3	1,408	3.04	1-2	153 - 2,000	519 - 1,916	0.46	0.59	0.51 ± 0.021	0.55 ± 0.035
83	Kinase	Tyrosine-protein kinase SRC	4,183	3.39	1-2	89 - 2,031	550 - 1,975	0.47	0.83	0.53 ± 0.023	0.79 ± 0.019
84	Kinase	Tyrosine-protein kinase receptor FLT3	2,274	3.50	1-2	101 - 1,617	833 - 1,988	0.48	0.74	0.53 ± 0.023	0.70 ± 0.019
85	Kinase	Epidermal growth factor receptor erbB1	7,122	3.24	1-2	159 - 1,878	791 - 1,957	0.48	0.80	0.55 ± 0.016	0.77 ± 0.011
86	Kinase	Serine/threonine-protein kinase AKT	3,114	3.59	1-2	97 - 1,912	824 - 1,887	0.48	0.77	0.53 ± 0.022	0.74 ± 0.022
87	Kinase	MAP kinase p38 alpha	4,518	3.37	1-2	91 - 2,034	463 - 1,837	0.48	0.73	0.54 ± 0.017	0.69 ± 0.010

88	Kinase	Serine/threonine-protein kinase PIM2	2,406	2.93	1-2	171 - 1,790	590 - 1,910	0.48	0.72	0.54 ± 0.025	0.68 ± 0.026
89	Kinase	Tyrosine-protein kinase JAK3	3,225	3.15	1-2	227 - 1,430	781 - 1,926	0.49	0.77	0.54 ± 0.018	0.75 ± 0.023
90	Kinase	Vascular endothelial growth factor receptor 2	8,936	3.40	1-2	133 - 2,044	1,275 - 1,998	0.49	0.69	0.55 ± 0.012	0.65 ± 0.015
91	Kinase	MAP kinase ERK2	3,712	3.28	1-2	589 - 1,566	920 - 1,947	0.50	0.80	0.56 ± 0.013	0.78 ± 0.0055
92	Kinase	Cyclin-dependent kinase 2	2,352	2.87	1-2	74 - 1,508	1,025 - 1,893	0.51	0.74	0.56 ± 0.019	0.71 ± 0.018
93	Kinase	Serine/threonine-protein kinase Aurora-A	2,949	3.39	1-2	486 - 1,687	668 - 2,009	0.56	0.75	0.62 ± 0.024	0.72 ± 0.015
94	Kinase	Tyrosine-protein kinase ABL	2,249	3.17	1-3	201 - 1,741	522 - 2,047	0.56	0.83	0.63 ± 0.044	0.79 ± 0.029
95	Kinase	Glycogen synthase kinase-3 beta	2,702	2.76	1-2	64 - 1,715	894 - 1,802	0.61	0.58	0.67 ± 0.022	0.55 ± 0.023
96	Nuclear receptor	Thyroid hormone receptor alpha	461	1.56	1-2	109 - 441	277 - 508	0.38	0.82	0.40 ± 0.014	0.82 ± 0.0078
97	Nuclear receptor	Glucocorticoid receptor	2,293	3.26	1-2	273 - 1,982	728 - 2,034	0.48	0.78	0.53 ± 0.026	0.74 ± 0.021
98	Nuclear receptor	Peroxisome proliferator-activated receptor gamma	3,018	3.32	1-2	192 - 1,412	951 - 2,015	0.51	0.72	0.55 ± 0.015	0.69 ± 0.010
99	Nuclear receptor	Vitamin D receptor	546	1.91	1-2	39 - 366	234 - 464	0.51	0.88	0.54 ± 0.030	0.87 ± 0.0095
100	Nuclear receptor	Androgen Receptor	2,597	1.68	1-2	193 - 1,865	1,295 - 1,942	0.56	0.72	0.60 ± 0.024	0.70 ± 0.019
101	Nuclear receptor	Estrogen receptor alpha	2,878	2.83	1-2	61 - 1,188	746 - 1,494	0.57	0.76	0.62 ± 0.012	0.74 ± 0.0043
102	Nuclear receptor	Estrogen receptor beta	2,229	2.59	1-2	358 - 1,503	584 - 1,666	0.67	0.68	0.72 ± 0.023	0.65 ± 0.023
103	Protease	Cathepsin D	2,568	3.29	1-2	59 - 1,946	952 - 1,968	0.39	0.85	0.42 ± 0.018	0.83 ± 0.0056
104	Protease	Matrix metalloproteinase-1	3,746	2.49	1-2	79 - 2,043	915 - 1,868	0.42	0.81	0.47 ± 0.020	0.78 ± 0.015
105	Protease	ADAM17	2,410	3.15	1-2	151 - 1,434	703 - 1,976	0.42	0.89	0.47 ± 0.022	0.87 ± 0.0084
106	Protease	Cathepsin S	2,309	3.44	1-2	104 - 1,386	1,152 - 2,025	0.46	0.79	0.50 ± 0.010	0.76 ± 0.013
107	Protease	Thrombin	6,703	3.50	1-2	220 - 1,779	1,399 - 1,894	0.49	0.84	0.56 ± 0.023	0.81 ± 0.014
108	Protease	Beta-secretase 1	7,554	3.17	1-2	295 - 1,934	737 - 1,971	0.50	0.74	0.57 ± 0.028	0.69 ± 0.025
109	Protease	Dipeptidyl peptidase IV	4,517	2.36	1-2	108 - 1,793	762 - 2,015	0.53	0.77	0.58 ± 0.010	0.74 ± 0.0082
110	Protease	Matrix metalloproteinase 13	2,844	2.83	1-2	161 - 976	746 - 1,820	0.54	0.74	0.59 ± 0.010	0.72 ± 0.0079
111	Protease	Trypsin I	2,501	3.34	1-2	202 - 1,979	630 - 1,934	0.57	0.91	0.63 ± 0.050	0.89 ± 0.018
112	Protease	Leukocyte elastase	2,808	2.64	1-2	239 - 1,702	923 - 1,984	0.57	0.93	0.63 ± 0.038	0.92 ± 0.017
113	Protease	Matrix metalloproteinase-2	4,085	2.76	1-2	133 - 1,732	434 - 2,039	0.58	0.81	0.60 ± 0.027	0.79 ± 0.020
114	Protease	Renin	3,010	3.64	1-2	82 - 1,840	536 - 1,916	0.58	0.74	0.62 ± 0.013	0.72 ± 0.010
115	Protease	Matrix metalloproteinase 9	3,149	2.65	1-2	240 - 1,503	210 - 1,824	0.59	0.70	0.65 ± 0.017	0.67 ± 0.010
116	Protease	Coagulation factor X	6,307	3.49	1-2	310 - 1,191	535 - 1,952	0.64	0.80	0.70 ± 0.023	0.77 ± 0.0079
117	Protease	Human immunodeficiency virus type 1 protease	4,571	3.65	1-2	147 - 1,032	901 - 1,997	0.94	0.65	1.04 ± 0.029	0.63 ± 0.018
118	Transporter	Potassium-transporting ATPase	532	1.58	1-2	207 - 501	221 - 496	0.40	0.52	0.42 ± 0.0081	0.53 ± 0.018
119	Transporter	GABA transporter 1	576	2.76	1-2	22 - 334	179 - 509	0.44	0.86	0.47 ± 0.040	0.84 ± 0.025
120	Transporter	Dopamine transporter	5,908	2.20	1-2	318 - 1,930	691 - 2,033	0.48	0.76	0.54 ± 0.014	0.72 ± 0.017
121	Transporter	Norepinephrine transporter	4,342	1.91	1-2	166 - 1,713	1,048 - 1,954	0.50	0.70	0.55 ± 0.015	0.66 ± 0.018
122	Transporter	Serotonin transporter	7,886	2.73	1-2	178 - 1,129	677 - 1,806	0.51	0.75	0.56 ± 0.011	0.72 ± 0.0094
123	Others	Histone deacetylase 1	4,239	2.49	1-2	198 - 2,022	1,533 - 2,008	0.41	0.74	0.47 ± 0.015	0.71 ± 0.017
124	Others	Bromodomain-containing protein 4	2,208	2.87	1-2	424 - 1,484	477 - 1,470	0.41	0.82	0.46 ± 0.032	0.79 ± 0.026
125	Others	Histone deacetylase 6	2,725	1.97	1-2	119 - 1,920	605 - 1,926	0.42	0.82	0.47 ± 0.023	0.79 ± 0.015
126	Others	p53-binding protein Mdm-2	2,346	3.51	1-2	33 - 1,936	1,277 - 1,950	0.42	0.88	0.47 ± 0.020	0.86 ± 0.011
127	Others	Sigma opioid receptor	3,094	2.53	1-2	48 - 1,366	502 - 1,814	0.53	0.56	0.58 ± 0.021	0.54 ± 0.019

a: Target names extracted from ChEMBL (release 25).

b: Data with the standard relation of "=" and pIC_{50} >= 0 were included in the present study.

c: MAE±standard deviation.

d: R^2±standard deviation.

Table S4. Average RMSE values for 25 targets

Target[a]	CNN[b]	RF[b,c]	FNN[b,c]	GCN[d]: KekuleScope dataset	GCN[d]: present dataset
Acetylcholinesterase	0.77	0.71	0.72	0.78	0.45
Alpha-2a adrenergic receptor	0.78	0.72	0.80	0.70	N.D.
Androgen Receptor	0.69	0.59	0.64	0.66	0.48
Cannabinoid CB1 receptor	0.79	0.71	0.74	0.74	0.37
Carbonic anhydrase II	0.69	0.60	0.61	0.58	0.57
Caspase 3	0.64	0.54	0.59	0.63	N.D.
Cyclooxygenase-1	0.74	0.66	0.72	0.73	0.45
Cyclooxygenase-2	0.82	0.77	0.80	0.75	0.64
Dihydrofolate reductase	0.79	0.73	0.77	0.98	0.68
Dopamine D2 receptor	0.78	0.71	0.72	0.87	0.38
Epidermal growth factor receptor erbB1	0.77	0.69	0.73	0.73	0.54
Estrogen receptor alpha	0.72	0.65	0.67	0.74	0.56
Glucocorticoid receptor	0.63	0.59	0.62	0.67	0.50
Glycogen synthase kinase-3 beta	0.83	0.70	0.78	0.78	0.50
HERG	0.61	0.59	0.61	0.61	0.34
Tyrosine-protein kinase LCK	0.87	0.79	0.84	0.87	N.D.
Monoamine oxidase A	0.71	0.63	0.68	0.70	0.59
Mu opioid receptor	0.80	0.67	0.70	0.64	0.54
Norepinephrine transporter	0.75	0.68	0.69	0.69	0.40
Serine/threonine-protein kinase Aurora-A	0.84	0.74	0.80	0.78	0.67
Serine/threonine-protein kinase B-raf	0.65	0.58	0.59	0.70	0.32
Thrombin	0.91	0.79	0.83	0.85	0.43
Tyrosine-protein kinase ABL	0.86	0.76	0.80	0.82	0.54
Tyrosine-protein kinase JAK2	0.72	0.62	0.67	0.69	0.50
Vanilloid receptor	0.74	0.70	0.72	0.72	0.33
Mean RMSE	0.76	0.68	0.71	0.74	0.49
Standard deviation	0.078	0.070	0.076	0.091	0.11

a: Target names extracted from ChEMBL (release 25).

b: The average of the RMSE values reported by the authors of KekuleScope.

c: The result of the models trained on Morgan fingerprints of 2048 bits.

d: The average of the RMSE values obtained from the individual models.

N.D.: Not determined

Table S5. Performance comparison of ensemble models on SET1-3

Protein family	Target[a]	Number of compounds	SET1		SET2		SET3		SET1-3[b]	
			MAE: ensemble	R^2: ensemble	MAE: ensemble	R^2: ensemble	MAE: ensemble	R^2: ensemble	MAE: ensemble	R^2: ensemble
GPCR	Gonadotropin-releasing hormone receptor	1,726	0.62	0.94	0.60	0.95	0.64	0.94	0.62 ± 0.018	0.94 ± 0.0050
GPCR	Muscarinic acetylcholine receptor M2	2,135	0.62	0.83	0.60	0.84	0.59	0.84	0.60 ± 0.012	0.84 ± 0.0056
GPCR	Mu opioid receptor	8,904	0.58	0.73	0.56	0.74	0.57	0.74	0.57 ± 0.010	0.74 ± 0.0068
GPCR	Dopamine D2 receptor	11,632	0.50	0.67	0.50	0.66	0.50	0.67	0.50 ± 0.0033	0.66 ± 0.0035
Enzyme	Gamma-secretase	1,932	0.59	0.67	0.57	0.68	0.58	0.67	0.58 ± 0.010	0.67 ± 0.010
Enzyme	Cytochrome P450 19A1	2,196	0.54	0.73	0.54	0.73	0.50	0.77	0.53 ± 0.022	0.74 ± 0.021
Enzyme	Carbonic anhydrase II	5,808	0.53	0.76	0.51	0.76	0.52	0.76	0.52 ± 0.012	0.76 ± 0.0030
Enzyme	Acetylcholinesterase	9,737	0.51	0.71	0.50	0.72	0.50	0.71	0.50 ± 0.0058	0.72 ± 0.0035
Ion channel	Transient receptor potential cation channel subfamily A member 1	621	0.56	0.68	0.54	0.71	0.57	0.67	0.56 ± 0.015	0.68 ± 0.018
Ion channel	Voltage-gated potassium channel subunit Kv1.5	739	0.39	0.53	0.37	0.58	0.41	0.53	0.39 ± 0.020	0.55 ± 0.029
Ion channel	Sodium channel protein type IX alpha subunit	5,677	0.42	0.72	0.42	0.72	0.42	0.71	0.42 ± 0.0028	0.72 ± 0.0050
Ion channel	HERG	9,198	0.38	0.66	0.37	0.67	0.37	0.68	0.37 ± 0.0035	0.67 ± 0.0089
Kinase	Fibroblast growth factor receptor 3	1,408	0.46	0.59	0.45	0.62	0.46	0.61	0.46 ± 0.0049	0.61 ± 0.013
Kinase	Tyrosine-protein kinase ABL	2,249	0.56	0.83	0.56	0.83	0.57	0.83	0.56 ± 0.0077	0.83 ± 0.0025
Kinase	Epidermal growth factor receptor erbB1	7,122	0.48	0.80	0.48	0.80	0.48	0.80	0.48 ± 0.0031	0.80 ± 0.0023
Kinase	Vascular endothelial growth factor receptor 2	8,933	0.49	0.69	0.49	0.69	0.49	0.70	0.49 ± 0.0033	0.70 ± 0.0082
Nuclear receptor	Thyroid hormone receptor alpha	461	0.38	0.82	0.44	0.76	0.38	0.80	0.40 ± 0.036	0.79 ± 0.031
Nuclear receptor	Vitamin D receptor	546	0.51	0.88	0.44	0.90	0.48	0.90	0.48 ± 0.032	0.89 ± 0.012
Nuclear receptor	Estrogen receptor alpha	2,878	0.57	0.76	0.58	0.76	0.58	0.76	0.58 ± 0.0069	0.76 ± 0.0017
Nuclear receptor	Peroxisome proliferator-activated receptor gamma	3,018	0.51	0.72	0.50	0.72	0.51	0.70	0.50 ± 0.0057	0.71 ± 0.0086
Protease	Cathepsin S	2,309	0.46	0.79	0.44	0.80	0.45	0.80	0.45 ± 0.010	0.80 ± 0.0064
Protease	ADAM17	2,410	0.42	0.89	0.43	0.89	0.44	0.88	0.43 ± 0.013	0.89 ± 0.0058
Protease	Thrombin	6,703	0.49	0.84	0.46	0.85	0.49	0.84	0.48 ± 0.014	0.85 ± 0.0080
Protease	Beta-secretase 1	7,554	0.50	0.74	0.51	0.74	0.51	0.74	0.51 ± 0.0062	0.74 ± 0.0030
Transporter	Potassium-transporting ATPase	532	0.40	0.52	0.38	0.53	0.41	0.52	0.40 ± 0.016	0.53 ± 0.0076
Transporter	GABA transporter 1	576	0.44	0.86	0.39	0.88	0.45	0.83	0.43 ± 0.031	0.86 ± 0.023
Transporter	Dopamine transporter	5,908	0.48	0.76	0.47	0.76	0.47	0.77	0.48 ± 0.0083	0.76 ± 0.0056
Transporter	Serotonin transporter	7,886	0.51	0.75	0.49	0.76	0.50	0.76	0.50 ± 0.0084	0.76 ± 0.0054
Others	Bromodomain-containing protein 4	2,208	0.41	0.82	0.44	0.79	0.43	0.81	0.43 ± 0.011	0.81 ± 0.012
Others	p53-binding protein Mdm-2	2,346	0.42	0.88	0.42	0.88	0.45	0.87	0.43 ± 0.014	0.88 ± 0.0059
Others	Sigma opioid receptor	3,094	0.53	0.56	0.53	0.53	0.53	0.55	0.53 ± 0.0015	0.55 ± 0.016
Others	Histone deacetylase 1	4,239	0.41	0.74	0.41	0.75	0.42	0.74	0.41 ± 0.0024	0.74 ± 0.0045

a: Target names extracted from ChEMBL (release 25).

b: The average and standard deviation of the MAE values for SET1-3.
